# Inverse association of resistin with physical activity in the general population

**DOI:** 10.1371/journal.pone.0182493

**Published:** 2017-08-03

**Authors:** Itahisa Marcelino-Rodríguez, Delia Almeida Gonzalez, José Juan Alemán-Sánchez, Buenaventura Brito Díaz, María del Cristo Rodríguez Pérez, Fadoua Gannar, Santiago Domínguez Coello, Francisco J. Cuevas Fernández, Antonio Cabrera de León

**Affiliations:** 1 Unidad de Investigación de Atención Primaria y del Hospital Universitario Nuestra Señora de Candelaria, Santa Cruz de Tenerife, Spain; 2 Red de Investigación Cardiovascular del Instituto de Salud Carlos III, Madrid, Spain; 3 Unidad de Inmunología, Hospital Universitario Nuestra Señora de Candelaria, Santa Cruz de Tenerife, Spain; 4 Faculty of Sciences of Bizerte, Carthage University, Tunis, Tunisia; 5 Área de Medicina Preventiva y Salud Pública, Universidad de La Laguna, La Laguna, Spain; Universidad Pablo de Olavide, SPAIN

## Abstract

**Aim:**

Resistin is a cytokine related with inflammation and ischemic heart disease. Physical activity (PA) prevents chronic inflammation and ischemic heart disease. We studied the relationship of serum concentration of resistin with HDL cholesterol, a known biomarker of PA, and with different measures of PA, in a large sample of the general adult population in the Canary Islands.

**Methods:**

Cross-sectional study of 6636 adults recruited randomly. We analyzed the correlation of resistin and HDL cholesterol with PA (as metabolic equivalent level [MET]), and fitted the results with linear and logistic regression models using adjustment for age, alcohol consumption and smoking.

**Results:**

Mean resistin level was higher in women (p<0.001), correlated inversely with age, HDL cholesterol (p<0.001) and alcohol consumption (p<0.001 in men), and correlated directly with smoking (p<0.001). Resistin correlated inversely with the duration of leisure time PA (p<0.001), leisure time MET (p<0.001) and moderate leisure time PA (p<0.001), with some differences between sexes. Men (OR = 0.78 [0.61–0.99; p<0.05]) and women (OR = 0.75 [0.61–0.92; p<0.01]) in the upper quintile of leisure time PA had a lower risk of elevated resistin. In contrast, a high degree of sedentarism was associated with an increased risk elevated resistin in women (OR = 1.24 [1.04–1.47; p<0.05] and in men (OR = 1.40 [1.01–1.82; p<0.05]).

**Conclusions:**

In our sample of the general population, resistin was inversely associated with measures and levels of PA and HDL cholesterol. The association of resistin with PA was stronger than the association of HDL cholesterol with PA, making resistin a potentially useful biomarker of PA.

## Introduction

The balance between pro- and anti-inflammatory cytokine secretion is a key factor in an organism’s metabolism and immune responses, and has effects on angiogenesis, blood pressure and lipid metabolism. These factors in turn are related with cardiovascular disease [1,2]. Resistin is a pro-inflammatory protein synthesized in humans mainly by macrophages; the production of this protein is associated with the expression of pro-inflammatory mediators such as tumor necrosis factor alpha (TNF-α), interleukin 1 (IL-1) and interleukin 6 (IL-6) [3], as well as with pathophysiological conditions such as atherosclerosis and ischemic heart disease [4]. Specifically, resistin promotes the formation of foam cells in the vascular wall [5], where cholesterol crystals trigger inflammatory processes by activating the inflammasome [6,7], the protein platform responsible for the activation of inflammatory processes. The inflammasome has been shown to induce cell pyroptosis, an inflammatory process of programmed cell death [8].

Given that physical activity (PA) can have significant anti-inflammatory effects depending on the type of exercise [9, 10], the relationship between resistin, types of PA and known biomarkers of PA–such as HDL cholesterol–is a potentially fruitful area for research [11]. A systematic review of assays that studied physical exercise in patients with diabetes found no effect on resistin [12]; however, smaller studies have reported that serum resistin levels decrease after moderate exercise [13–17], but increase after vigorous exercise [18, 19], and that these changes are associated with markers of cartilage degradation [20]. It has been demonstrated that resistin correlates inversely with serum HDL cholesterol level in a small group of 34 patients with abdominal obesity [21]. This raises the possibility that studies in large population-based samples may provide conclusive evidence of the relationship between PA and resistin levels in people with different levels of PA (active and passive leisure activities, and light, moderate or vigorous PA), and different amounts of time at rest.

The aim of this study was to investigate the relationship of serum resistin with different types of PA and times at rest in a large sample of the general adult population, with adjustment for possible interactions with life style factors.

## Material and methods

The participants in this cross-sectional study were drawn from the *CDC de Canarias* cohort (CDC is the acronym for *C*ardiovascular, *D*iabetes y *C*áncer–the name of the study in Spanish), which was selected randomly during the years 2000 to 2005 from the general population in the Canary Islands aged between 18 and 75 years. The methodology has been described in detail elsewhere [22]. Participants recruited for this analysis (n = 6729) were interviewed to obtain responses to a questionnaire on their health-related antecedents (the instrument is available at *http://www.cdcdecanarias.org*) and life style (hours of nighttime sleep, daytime napping sleep and television watching, alcohol consumption [g/day] and smoking [g/day]). The study was approved by the Bioethics Committee of Nuestra Señora de la Candelaria University Hospital, and all participants provided their informed consent in writing.

A sample of venous blood was obtained after the participant had abstained from food or drink for at least 10 hours. All samples were centrifuged in situ at room temperature (2000 rpm for 10 min) and transported daily to the laboratory. Serum HDL cholesterol was measured with in a Hitachi 917 autoanalyzer within 24 hours after the sample was obtained, and the results were recorded as serum concentration in mg/dL. Serum samples were frozen and stored at −80°C, and one aliquot was thawed and used for resistin determination by enzyme immunoanalysis (ng/mL, Bio-Vendor®, Heidelberg, Germany; between-assay coefficient of variation, 7.72%; within-assay coefficient of variation, 2.22%).

Data on PA during leisure time were recorded with the Spanish version of the Minnesota Leisure Time Physical Activity Questionnaire [23, 24], and data on PA during work were obtained with a validated questionnaire for the Canary Islands population (daily hours of PA equivalent to or more vigorous than brisk walking). Each activity reported by the participants was assigned a metabolic equivalent level (MET). One MET reflects an individual’s energy consumption at rest, equivalent to approximately 1 kcal per kg body weight per hour, or 4.184 kJ per kg body weight per hour according to the Compendium of Physical Activities of Ainsworth et al. [25].

Passive leisure PA was considered any activity in which MET consumption was less than 4, and active leisure PA was considered any activity with a MET level equal to or higher than 4. Measurements of MET during leisure time did not consider usual housework activities. Leisure PA was classified in three categories designated light (MET <3.5), moderate (MET 3.5–6) or vigorous (MET >6). The level of sedentarism was measured as the quotient of daily MET for moderate leisure time PA divided by total daily leisure time MET. Total MET values were calculated as the sum of all MET levels attributed to sleep, work and leisure time. Leisure time PA was classified according to three criteria: daily MET for leisure time (≤4.6, 4.6–18 and >18 MET), MET for the time spent on PA in the week (≤45, >45–105 and >105 min), and the daily amount of moderate or vigorous PA (none, low or recommended).

We also collected data on some variables whose relationship with resistin had been previously analyzed in this population: Diabetes, hypertension, ischemic cardiopathy, obesity index (waist to height ratio) and Mediterranean diet adherence. The methods to obtain these variables have been described [4, 22, 23, and 26]. All data is available as supporting information (S1 Database).

### Statistical analysis

The associations of resistin and HDL cholesterol with different measures of PA were analyzed with Spearman’s nonparametric test. Group comparisons were done with analysis of variance. The variables were summarized as the mean ± standard deviation, and log transformation was used for resistin values to approximate them to a normal distribution.

The bivariate associations found in the initial analysis were adjusted with multivariate models using covariates found to be associated with resistin: age (years), alcohol consumption (g/day) and smoking consumption (g/day), plus diabetes, hypertension, ischemic cardiopathy, obesity index (waist to height ratio) and Mediterranean diet adherence. In the linear models we used different measures of PA performed in the previous week as the independent variable, and serum resistin (with log transformation) or HDL cholesterol concentration as the dependent variables. The strength of these associations was tested with a Z-test. For the logistic regression models, we categorized independent variables (for PA) and dependent variables (for resistin and HDL cholesterol) as within or below the 80th percentile (p80). The tables show the odds ratios with 95% confidence intervals (OR [95% CI]) and p values for the highest quintile versus the lowest quintile.

All calculations were done with SPSS version 21 software in Spanish.

## Results

The final sample in the present analysis comprised 6637 participants (3757 women; 57%). Mean resistin concentration was 6.06±2.41 ng/mL in women and 5.63±2.18 ng/mL in men, and mean HDL cholesterol concentration was 54.69±13.18 mg/dL and 46.42±12.30 mg/dL, respectively. These two biomarkers were inversely correlated (p<0.001). In both sexes resistin correlated inversely with age and directly with smoking (Table 1), but the inverse correlation between resistin and alcohol consumption was significant only in men (ro = −0.067; p<0.001). In contrast, HDL cholesterol correlated inversely with smoking and directly with alcohol consumption.

**Table 1:** Spearman nonparametric coefficients correlation of resistin and HDL cholesterol with different measures of physical activity.

	Resistin (ng/mL)		HDL Cholesterol (mg/dL)	
Final week	Women n = 3757	Men n = 2880	Women n = 3757	Men n = 2880
Age (years)	-0.182**	-0.106***	-0.103**	0.022
Alcohol consumption (g/day)	0.021	-0.067***	0.112***	0.160***
Smoking (g/day)	0.134***	0.119***	-0.105***	-0.088***
Hours of nighttime sleep	0.042**	-0.012	0.051**	-0.018
Hours of daytime sleep	0.021	-0.012	-0.020	-0.023
Hours of physical activity at work	-0.018	0.030	-0.007	0.021
MET physical activity at work	0.034*	-0.014	0.047**	0.047*
Hours of leisure time	-0.041*	-0.089***	-0.013	0.032
Hours of physical activity during leisure time	-0.041*	-0.104***	0.013	0.038*
MET during leisure time	-0.073***	-0.104***	0.046**	0.044*
MET during active leisure	-0.028‡	-0.075***	0.027	0.057**
MET during passive leisure	-0.032‡	-0.017	-0.021	-0.027
MET total (sleep, work, leisure time)	-0.016	-0.037*	0.014	0.081***
MET active (active work and active leisure time)	-0.015	-0.037*	0.034*	0.075***
MET leisure time light physical activity	-0.012	0.012	0.030‡	0.006
MET leisure time moderate physical activity	-0.068***	-0.114***	0.046**	0.036‡
MET leisure time vigorous physical activity	-0.010	0.027	0.025	0.056**
Sedentarism	0.078***	0.110***	-0.064***	-0.008
HDL Cholesterol	-0.060***	-0.072***

‡ p<0.10

*p<0.05

**p<0.01

***p<0.001.

METweek1 = Metabolic equivalent units during physical exercise during 1 week. Sedentarism CDC = Daily MET during active leisure activities / total daily MET

Table 1 shows the inverse correlation of resistin and the direct correlation of HDL cholesterol with different PA variables, stratified by sex. In men, the strongest association was seen for resistin with hours of leisure time PA, leisure time MET (both variables; r = −0.104; p<0.001), and sedentarism (r = −0.110; p<0.001). More specifically, resistin showed an inverse correlation with MET for moderate leisure time PA (r = −0.114; p<0.01). In women, these correlations were weaker but were in the same direction. Sedentarism correlated directly with resistin concentration, and inversely with HDL cholesterol concentration.

Table 2 summarizes the associations of resistin and HDL cholesterol with different categories of leisure time PA. Only resistin had significant associations in both sexes with different PA variables (e.g. duration of PA, MET level and intensity of PA). In contrast, the associations for HDL cholesterol did not reach statistical significance in women at any MET level during leisure time (p = 0.093), or in men at any intensity level of leisure time PA (p = 0.235).

**Table 2:** Distribution of resistin and HDL cholesterol across categories of leisure time physical activity, stratified by sex.

	Resistin (ng/mL)		HDL Cholesterol (mg/dL)	
	Women	Men	Women	Men
Leisure time ≤4.6 MET/day	6.17±2.41 n = 2131	5.79±2.19 n = 1192	54.28±13.17 n = 2131	45.75±12.20n = 1192
Leisure time >4.6 y ≤ 18 MET/day	5.96±2.43 n = 916	5.71±2.25 n = 739	55.19±13.25 n = 916	46.68±11.99n = 739
Leisure time >18 MET/day	5.89±2.39 n = 710	5.38±2.11 n = 949	55.27±13.13 n = 710	47.07±12.64n = 949
	p = 0.002*	p <0.001*	p = 0.093	p = 0.039
Active leisure time ≤45 min/Total leisure time	6.15±2.41 n = 2084	5.79±2.18 n = 1681	54.27±13.15 n = 2084	45.95±11.94n = 1681
Active leisure time >45 y ≤105 min/Total leisure time	6.05±2.62 n = 819	5.45±2.20 n = 530	55.70±13.07 n = 819	47.02±11.36n = 530
Active leisure time >105 min/Total leisure time	5.86±2.21 n = 854	5.39±2.15 n = 669	54.74±13.34 n = 854	47.15±13.78n = 669
	p = 0.014*	p <0.001*	p = 0.031	p = 0.048
No MVPA	6.19±2.45 n = 1756	5.79±2.15 n = 997	53.90±13.18 n = 1756	45,90±12,06 n = 997
Low MVPA	6.02±2.45 n = 1302	5.62±2.27 n = 1351	55.59±12.95 n = 1302	46,63±12,00 n = 1351
Recommended MVPA	5.81±2.23 n = 699	5.36±1.99 n = 532	54.99±13.53 n = 699	46,87±13,43 n = 532
	p = 0.001*	p <0.001*	p = 0.002	p = 0.235

*For both, resistin and HDL cholesterol, comparisons were made between serum concentrations of men or women in relation to three categories of physical activity (ANOVA).

For resistin, the tests were applied after natural log-transformation of this serum biomarker. MVPA: Moderate to vigorous physical activity.

Table 3 shows the results of multivariate linear regression analysis. Associations were found for both resistin and HDL cholesterol with PA, and although adjustment weakened the strength of some of the bivariate associations, it did not affect their direction. Table 4 summarizes the OR and 95% CI obtained with multivariate logistic regression models to adjust the association between resistin values in the 80th percentile (dependent variable p80 = 7.28 ng/mL in women and 6.73 ng/mL in men) and PA values in the 80th percentile. In women, only participants in the 80th percentile for leisure time spent on PA (>20 min/day) showed a significantly lower risk of belonging to the 80th percentile for resistin levels (OR = 0.75; p<0.01). Participants in the 80 the percentile for sedentarism had a higher risk of elevated resistin levels in men (OR = 1.27). In women, other variables showed a significant protective effect (leisure time passive PA MET), whereas an increased risk for elevated resistin was found in women in the 80th percentile for napping time (OR = 1.25; p<0.05).

**Table 3:** Standardized regression coefficients obtained in linear regression models.^$^.

	Resistin (ng/mL)		HDL Cholesterol (mg/dL)	
INDEPENDENT VARIABLE	Women n = 3757	Men n = 2880	Women n = 3757	Men n = 2880
Hours of nighttime sleep	0.002	-0.026	0.003	-0.033^‡^
Hours of daytime sleep	0.033*	0.028	-0.006	-0.021
Hours of physical activity at work	0.010	0.030	-0.006	0.028
MET physical activity at work	0.007	-0.010	0.005	0.035
Hours of leisure time	-0.018	-0.033	0.003	0.020
Hours of physical activity during leisure time	-0.032^‡^	-0.048*	0.011	0.010
MET during leisure time	-0.024	-0.038*	0.017	0.030^‡^
MET during active leisure	-0.002	-0.033^‡^	0.011	0.038*
MET during passive leisure	-0.015	-0.016	-0.001	-0.019
MET total (sleep, work, leisure time)	-0.003	-0.018	0.010	0.038*
MET active (active work and active leisure time)	0.005	-0.011	0.009	0.038*
MET leisure time light physical activity	-0.007	0.017	-0.006	-0.006
MET leisure time moderate physical activity	-0.029^‡^	-0.049**	0.021	0.028
MET leisure time vigorous physical activity	0.003	0.011	0.007	0.030^‡^
Sedentarism	-0.039*	-0.073**	0.047**	0.001
HDL cholesterol	-0.076***	-0.042*

^$^ Each line offers the standardized coefficients of the independent variable in four models: Two models for resistin (One model for men and one for women), and two for HDL cholesterol (Also, one model for men and one for women). In each model the dependent variable was resistin or HDL cholesterol, as indicated, and the independent variable was adjusted by age, alcohol consumption, smoking, diabetes, hypertension, ischemic cardiopathy, obesity index (waist to height ratio), and Mediterranean diet adherence.

^‡^p<0.10

*p<0.05

**p<0.01

***p<0.001

**Table 4:** Each line offers the odds ratio of the independent variable in four logistic models: Two models for resistin (one model for men and one for women), and two for HDL cholesterol (also, one model for men and one for women). In each model the dependent variable was resistin (p80) or HDL cholesterol (p80), as indicated, and the independent variable was adjusted by age, alcohol consumption and smoking; these models also adjusted for diabetes, hypertension, ischemic cardiopathy, obesity index (waist to height ratio) and Mediterranean diet adherence.

	Resistin p80		HDL Cholesterol p80	
INDEPENDENT VARIABLE	Women n = 3757 OR (95% CI)	Men n = 2880 OR (95% CI)	Women n = 3757 OR (95% CI)	Men n = 2880 OR (95% CI)
P_80_ Hours of nighttime sleep	1.05 (0.89–1.25)	0.85 (0.69–1.04)	0.98 (0.82–1.16)	0.87 (0.71–1.06)
P_80_ Hours of daytime sleep	1.25 (1.03–1.52)*	1.12 (0.90–1.41)	1.04 (0.85–1.27)	0.96 (0.76–1.20)
P_80_ Hours of physical activity at work	0.93 (0.75–1.15)	0.95 (0.78–1.16)	0.84 (0.68–1.04)	1.01 (0.82–1.23)
P_80_ MET physical activity at work	1.00 (0.84–1.19)	0.98 (0.79–1.22)	1.11 (0.93–1.31)	1.29 (1.04–1.59)*
P_80_ Hours of leisure time	0.83 (0.66–1.03) ‡	0.83 (0.65–1.06)	1.10 (0.89–1.36)	0.99 (0.79–1.25)
P_80_ Hours of physical activity during leisure time	0.75 (0.61–0.93)**	0.80 (0.63–1.02)‡	1.16 (0.95–1.41)	0.96 (0.77–1.21)
P_80_ MET during leisure time	0.88 (0.71–1.09)	0.89 (0.70–1.14)	1.23 (1.00–1.50)*	0.97 (0.77–1.22)
P_80_ MET during active leisure	0.95 (0.77–1.17)	1.05 (0.853–1.33)	1.10 (0.90–1.35)	1.19 (0.95–1.48)
P_80_ MET during passive leisure	0.79 (0.64–0.98)*	0.92 (0.72–1.16)	1.21 (0.99–1.48) ‡	0.95 (0.75–1.19)
P_80_ MET total (sleep, work, leisure time)	0.81 (0.66–1.00) ‡	1.03 (0.82–1.30)	1.13 (0.93–1.38)	1.25 (1.00–1.56) ‡
P_80_ MET active (active work and active leisure time)	0.97 (0.79–1.19)	1.00 (0.79–1.26)	1.14 (0.94–1.40)	1.22 (0.95–1.52) ‡
P_80_ MET leisure time light physical activity	0.83 (0.66–1.03) ‡	0.94 (0.74–1.21)	1.12 (0.91–1.38)	1.22 (0.97–1.54) ‡
P_80_ MET leisure time moderate physical activity	0.88 (0.71–1.08)	0.83 (0.65–1.05)	1.22 (1.00–1.49)*	0.96 (0.76–1.20)
P_80_ MET leisure time vigorous physical activity	0.86 (0.70–1.06)	0.94 (0.76–1.17)	1.27 (1.04–1.54)*	1.16 (0.94–1.42)
P_80_ Sedentarism	0.99 (0.80–1.22)	1.27 (1.02–1.58)*	0.75 (0.61–0.92)**	1.03 (0.84–1.27)
P_80_ HDL cholesterol	0.76 (0.61–0.93)*	0.83 (0.65–1.05)

‡p<0.1

*p<0.05

**p<0.01.

In women (Table 4), the risk of inclusion in the 80th percentile for serum HDL cholesterol concentration was increased only if values for PA during leisure time were also in the 80th percentile, was the activity moderate (OR = 1.22), or intense (OR = 1.27). In contrast, sedentarism (OR = 0.75; p<0.01) was associated with a lower likelihood of elevated HDL cholesterol. In men, a significant risk of elevated HDL cholesterol was associated only with values in the 80th percentile of PA at work (OR = 1.29; p<0.05).

## Discussion

We confirmed the inverse association between resistin and PA, and between the former and HDL cholesterol, in the general adult population. Multivariate models with adjustment for factors that can modify resistin levels corroborated that this cytokine decreases as the time spent on leisure time PA increases, particularly in persons with moderate PA. The risk of elevated serum resistin concentrations was lower in persons who spent more than 20 min/day on PA during their leisure time, whereas this risk was higher in those with a more sedentary life style.

To our knowledge this is largest sample of the general population in which the relationship between PA and resistin has been studied with data for both measures. We previously reported in this population a direct association between resistin and the risk of ischemic heart disease, [4] and between this cytokine and saturated fat intake and serum triglyceride concentration [26]. The present study provides novel information in support of the notion that resistin can be considered a marker of cardiovascular risk. Our results suggest that using resistin as a biomarker for PA is potentially useful and may have clinical advantages compared to HDL cholesterol, which is influenced by variations in the relative proportions of its different subfractions [27].

Free fatty acid levels and serum lipopolysaccharides are involved in the relationship between PA and resistin. Physical exercise affects serum levels of these lipids, and the signals they transmit are involved in activation of the innate immune system. The mechanisms by which exercise affects innate immunity can be explained by factors such as oxidative stress, increased metabolic rate, heat shock proteins, catecholamines, corticosteroids and insulin-like growth factor, which can alter the expression of recognition molecules such as toll-like receptors [28].

The intensity of PA influences how biomarker concentrations are modified. The relationship between PA and health has been modeled as the well-known U-shaped curve, which illustrates that PA at moderate, regular doses has beneficial effects whereas excessive or vigorous PA can have negative consequences by triggering inflammatory reactions [29]. Moderate exercise is known to lower free fatty acid and lipopolysaccharide levels, which in turn reduces the tissue activation of toll-like receptor 4 (TLR4) and nuclear factor kappa B (NF-κB) [30, 31]. Under these circumstances, levels of lipopolysaccharides from the intestine are low, and in Kupffer cells an escape mechanism is triggered which involves IL-10 and suppresses pro-inflammatory cytokines. In contrast, sustained vigorous exercise leads to an increase in free fatty acids as a result of their mobilization from adipose tissue to provide energy to skeletal muscles; in this situation TLR4 activation and the NF-κB pathway are stimulated [32]. In addition, intense exercise increases intestinal permeability and serum lipopolysaccharide concentrations [33]; high concentrations lead to massive stimulation of TLR4 in Kupffer cells, which undergo classic polarization (M1) characterized by the production of inflammatory cytokines and reactive oxygen species. Resistin may stimulate polarization toward the M1 phenotype, in light of earlier research that reported an association between enhanced polarization and intense exercise [34]. Moderate PA, which we show here to be associated with lower serum resistin levels, can stimulate the alternative macrophage polarization phenotype (M2) characterized by anti-inflammatory functions and associated with cytokines that have the opposite effect to resistin, such as adiponectin [35]. Our results, which are consistent with earlier findings, show that the level of sedentarism was clearly a risk factor for elevated resistin and lower HDL cholesterol levels. In a small group of patients with abdominal obesity HDL cholesterol correlated inversely not only with resistin but also visfatin, and TNF-α. A possible functional mechanism for PA is to induce an HDL upregulation of scavenger receptors in adipocytes, which may suppress free cholesterol accumulation and prevent adipocyte inflammation [21].

Our results for duration of nighttime or daytime sleep showed that the time spent napping was directly associated with serum resistin levels, and that participants in the 80th percentile of napping time had a risk of being in the 80th percentile of resistin level, although in the multivariate analysis these associations were significant only in women. This can be attribute to the inverse association of napping time with MET spent in leisure time (data not shown, but available in the database included as a supplemental file). The direct association between resistin and hours of sleep was reported previously in a study of a small sample of young women [36]; however, we were unable to confirm this association for daytime sleep time, possibly because of we did not analyze dietary composition–a factor known to interact with measures of resistin [26] and the duration of sleep [37]. One earlier study reported that in addition to the duration of sleep, another factor that can affect serum cytokine concentrations is circadian rhythm misalignment, which increases IL-6, C-reactive protein, resistin and TNFα levels, in addition to increasing blood pressure and the risk of cardiovascular disease [37]. Moreover, one recent study reported an association of sleep duration (both insufficient sleep and excessive sleep) with insulin sensitivity and pancreatic beta cell function, and that this association differed between sexes [38]. The possible associations between these factors were not analyzed in the present study.

Among the limitations of our study, we note firstly that the cross-sectional design does not allow us to infer causal relationships between the measures we analyzed; only associations can be demonstrated. Nevertheless, the probable unidirectional nature of the association between PA and resistin together with current knowledge of the association between PA and HDL cholesterol support a similarly unidirectional association between resistin and these two variables. There will be always much more information that could be introduced as variables to adjust for (medication, arthritis, etc.) but, to our knowledge, there is not any described factor that can influence resistin in the general population and modify our results. The decision to exclude PA during housework may have influenced the associations we found, especially in women in our cultural setting; however we also tested the variable including the household work (data not shown, but available in the database included as a supplemental file) and the results were weaker than excluding it. The main strength of our study is the large size of our sample of the general population which provided data on serum resistin concentrations.

In conclusion, serum resistin concentration was inversely associated with physical activity in the sample of the general adult population we studied. Resistin is an indicator of physical activity that showed stronger associations than HDL cholesterol with different measures and levels of sedentarism and leisure time physical activity. Thus, resistin is potentially useful in clinical settings as a biomarker of physical activity in efforts to improve health.

## Supporting information

S1 Database (SAV)
